# Enhancing urban vehicular communication and safety through HMM-OCR

**DOI:** 10.1038/s41598-026-42007-y

**Published:** 2026-03-14

**Authors:** Sumadeep Juvvalapalem, Vadivukkarasi Kanagaraj

**Affiliations:** Department of Electronics and Communication Engineering, College of Engineering and Technology, SRM Institute of Science and Technology, Kattankulathur, Tamilnadu India

**Keywords:** VANETs, Routing, Infrastructure, Communication, Ad-hoc networks, Intelligent transportation, Engineering, Mathematics and computing

## Abstract

Vehicular Ad Hoc Networks (VANETs) work in urban environments where the topology changes due to high mobility, limited communication and dense traffic conditions. These factors lead to increase in end-to-end delay, energy consumption, and significant packet loss such challenges highlight the need for robust and adaptive routing mechanisms that can maintain reliable communication under dynamic and dense traffic scenarios. To overcome these issues, this study proposes a Hybrid Meta-Heuristic and Machine Learning-based Optimised Cluster-Based Routing (HMM-OCR) aimed at enhancing communication reliability and routing efficiency in urban VANETs. The proposed method integrates Modified Golden Eagle Optimisation (MGEO) for energy efficient clustering and Improved Jackal Optimisation (IJO) for optimal cluster head selection. Additionally, a Multivariable Output Neural Network (MONN) is employed to ensure efficient data forwarding and path establishment. Simulation results obtained using NS2 shows that HMM-OCR outperforms across key performance metrics. Specifically, HMM-OCR enhances throughput by 4.83–8.55%, reduces packet drop by 4.53–22.24%, improves packet delivery by 3.78–21.9%, reduces delay by 0.56–2.24 s, energy consumption by 20.38–62.43%, and routing overhead by 5.87–22.87%. These results clearly demonstrate that HMM-OCR method which significantly, enhances communication efficiency and reliability in urban environments, making it suitable for intelligent transportation systems.

## Introduction

Vehicle Ad Hoc Networks (VANETs), an important part of Intelligent Transportation Systems (ITS), and are mainly used to improve traffic flow, road safety, and driver comfort^1,2^. VANETs enable real-time communication between vehicles to share information related to traffic conditions, possible hazards, and road conditions through short-range wireless communication. The main objective of VANETs is to enhance traffic safety and efficiency by traffic congestion, accidents, and other travel-related issues^3,4^. In high density environments, traditional traffic control methods rely on techniques such as voice alerts, gestures, vehicle horns, and vehicle trajectory tracking to manage traffic flow^5^.

Recent technological advancements focus on reducing traffic congestion by introducing automated traffic signal controllers and intelligent vehicle indicators. However, despite these advancements, nearly 1.3 million accidents still occur worldwide annually, which is serious concern. Intelligent Transportation System (ITS) plays a crucial role in addressing such issues by predicting accident prone locations in advance. These systems use variable message signs to share real-time information and ensure effective coordination and management of Road Side Units (RSUs).

VANET offers a wide range of services like collision warnings, driving guidance, localization of vehicle, parking assistance, and automatic steering control. Protocols in VANETs are categorized to address the unique challenges includes: unicast, geo cast, multicast, hybrid, traffic aware routing, and geographic routing^6–8^.

### Metaheuristic algorithms

By dynamically optimising route selection based on variables such as traffic, link quality, and energy efficiency, metaheuristic algorithms improve VANET routing. In the dynamic environments of VANETs, their flexibility boosts packet delivery, throughput, lowers latency, and improves overall network performance.

The technique dynamically modifies beacon intervals to optimise network performance, thereby lowering the total number of messages and beacons sent^9^. Regin et al. introduced an algorithm in^10^ that uses node density based on received signal to avoid congestion. High traffic volumes and scarce network resources cause congestion, which is addressed by the suggested method. With an average PDR increase of 11.35% and a cluster overhead reduction of 9% in the sparse scenario, the method outperformed the current approach. In comparison to the current method, the PDR increased by 2%, the E2E delay decreased by 13%, and the cluster overhead selection decreased by 19% during the dense scenario.

### Hybrid algorithms

Because they combine proactive and reactive tactics, hybrid algorithms are essential to VANET. These algorithms adjust to the ever-changing landscape of vehicular communication. These algorithms improve security and privacy in VANETs, optimise energy consumption, and handle various QoS requirements. Because of this, hybrid algorithms are ideally adapted to the particular difficulties associated with dependable and effective vehicle communication.

Rajendra Mani et al. presented a hybrid algorithm with an integration of seagull and thermal exchange optimization to identify non-line of sight vehicles. The proposed method demonstrated notable performance gains, achieving a 14.86% increase in PDR, a 13% improvement in neighborhood awareness rate, and an 11.24% enhancement in channel utilization compared to existing approaches^11^. Ahmad et al.^12^ combined two optimization algorithms, honey bee, and genetic algorithms, to create efficient clusters in VANETs. The method achieved average of E2E delay reduced by10%, VANET life time increased by 48% approximately, and number of clusters required reduced by 3% with respect to existing methods. Gagan deep singh et al. presented an integration of genetic and firefly algorithm for faster communication and outperforms firefly and Particle Swarm Optimization with 0.77% and 0.55% of significance in dense network and 0.74% and 0.4% in sparse network scenarios^13^.

The contributions of this work are directed toward enhancing communication reliability and coordination in urban Vehicular Ad Hoc Networks through an optimized cluster-based routing framework termed HMM-OCR. Unlike existing hybrid VANET approaches that employ metaheuristic optimization and machine learning in isolated or loosely coupled stages, the proposed method introduces a tightly integrated optimization–learning architecture. Specifically, a Modified Golden Eagle Optimization (MGEO) algorithm and an Improved Jackal Optimization (IJO) algorithm are jointly employed for coordinated cluster formation and cluster head selection. Furthermore, a Multivariable Output Neural Network is utilized for adaptive data dissemination and path establishment rather than standalone prediction. The use of static road map based geographic routing, along with methods to improve stability of clusters and avoid frequent re-clustering makes the proposed approach more effective. As a result, it improves throughput, packet delivery, energy efficiency, overhead, and reduces delay and maintains overall network stability.

The paper’s remaining structure is set up as follows: The problem statement and network model are discussed in Section II. Section III explains the proposed methodology. Section IV discusses the results of the simulation. Future research and a conclusion are included in Section V.

## Problem statement and network model of HMM-OCR

### Problem statement

Arbelo Lolai et al.^14^ introduced a reinforcement learning protocol for efficient data dissemination in VANETs, utilizing MATLAB as their primary tool. Their strategy demonstrated enhanced system performance and flexibility in changing conditions. Their approach did have some drawbacks, though, most notably the intricacy of the computation process. Their study was primarily concerned with assessing PDR and delay time. Routing protocols depend on a structure’s probability of meeting some but not all of the requirements. Considerable progress has been made in offering effective and flexible solutions to prevent issues on the roads. In order to evaluate the effectiveness of different filtering mechanisms, experts have mostly concentrated on particular VANET features, such as cities or interstates. Managing obstructions in congested areas, which can disrupt the network’s architecture, is one of the primary difficulties these protocols encounter.

**Fig. 1:**
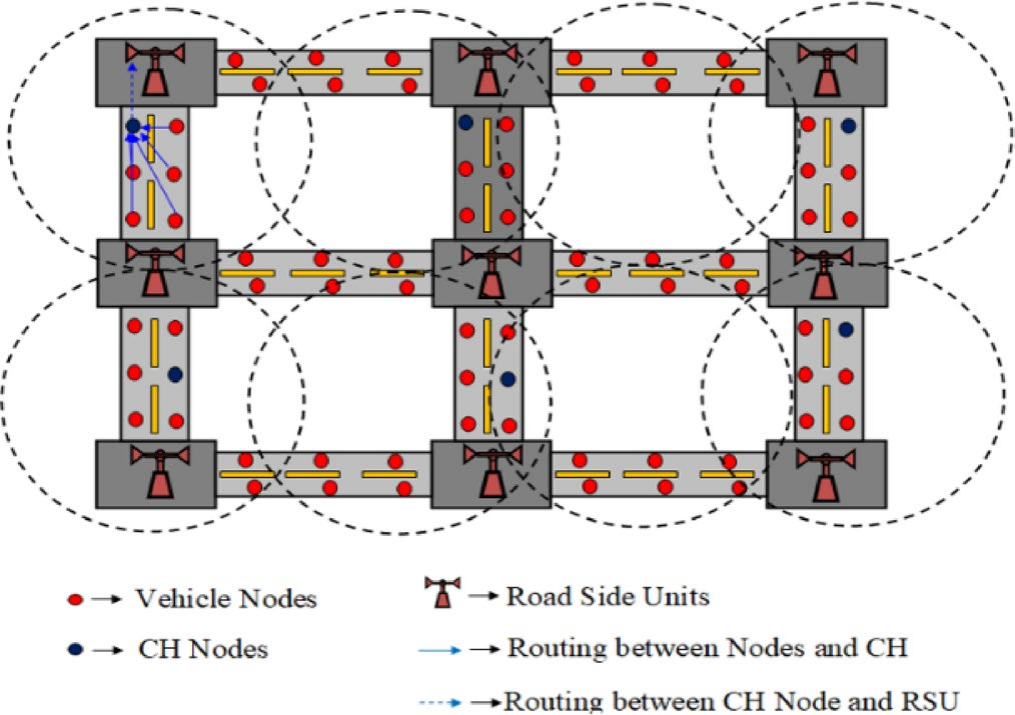
HMM-OCR proposed network model.

From the source to the destination, the transmission of data frequently takes lengthy paths through clusters, which results in delays and high energy consumption. Additionally, this may result in superfluous clusters and complicate overhead management. Another major problem is mobility, which makes it difficult to design effective routes, as Fig. 1 illustrates. In relation to this, the network model of our suggested approach, in which cars serve as nodes, is shown in Fig. 1. Cluster heads (CHs) are selected based on multiple factors, and road side units (RSUs) help in distributing data among vehicles, which further increases the complexity of routing. To address these challenges, this work builds upon existing studies and extends their scope by considering additional performance metrics such as energy consumption, packet drop ratio, routing overhead, packet delivery ratio (PDR), and end-to-end (E2E) delay.

The proposed HMM-OCR approach follows a three-stage process to evaluate these metrics. This extension provides a more comprehensive analysis of VANET performance and ensures a detailed assessment of their operational efficiency and reliability.

We intend to build on their work and expand the scope in order to overcome these challenges by considering additional performance metrics such as energy consumption, packet drop ratio, routing overhead, PDR, and E2E delay when adopting HMM-OCR using a threefold process. This extension aims to provide a more complete understanding and improvement of VANETs, ensuring a thorough assessment of their operational efficiency and reliability.

The following is the primary goal of the suggested HMM-OCR technique:

- Enhancing coordination and communication by incorporating the advantages of geographic routing for effective and trustworthy data distribution.
- Cluster formation using modified golden eagle optimization (MGEO) which ensures energy efficient data transmission by grouping vehicles into clusters based on their proximity and optimizing the cluster formation process.
- Cluster head (CH) selection with improved jackal optimization (IJO) by considering various design constraints to identify the most suitable CHs, which plays a crucial role in coordinating communication within the clusters.
- Complete path establishment with multivariable output neural network (MONN) which facilitates the data dissemination with infrastructure, overcoming challenges caused by obstacles.
- HMM-OCR executed in diverse environments, and the simulation outcomes shows that it performs better than the existing protocols.

### Network model of HMM-OCR

**Fig. 2:**
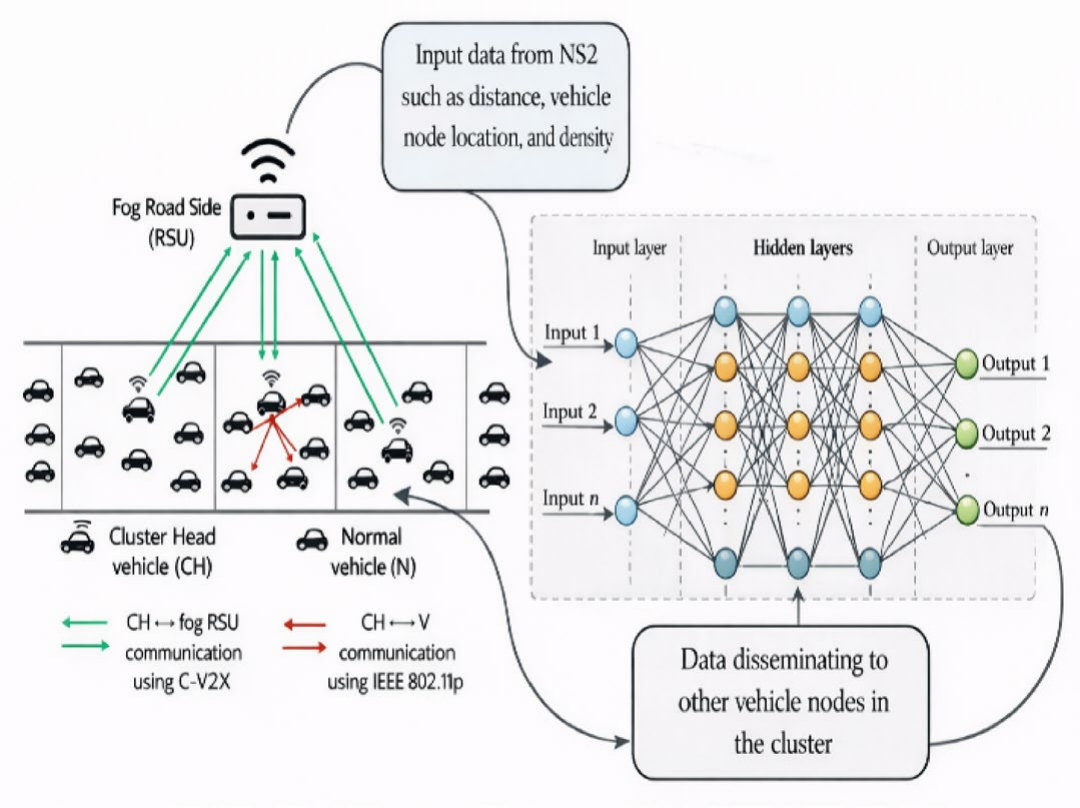
Proposed HMM-OCR method.

The VANET environment comprises vehicles as nodes along with RSUs. Figure 1 illustrates the network model of the proposed HMM-OCR and Fig. 2 represents the proposed HMM-OCR, wherein data trained by a neural network, receiving inputs from RSUs comprising vehicle density, distance, and location, is transmitted to neighboring vehicles to ensure collision avoidance and safe passage. In terms of contribution, the initial step involves completing efficient cluster formation, followed by the selection of Cluster Heads (CH). Subsequently, an efficient neighborhood node is computed for data dissemination between the source vehicle and destination through RSU. To maximise system performance and address metrics, the three-step procedure is carried out concurrently in order to improve road safety and encourage effective transportation.

## Proposed HMM-OCR method

This section describes the HMM-OCR method using a three-step sequential process. At first, we explain how cluster are formed followed by CHs considering several constraints to ensure the approach is complete.

### Selection of cluster using modified golden eagle optimization

Modified Golden Eagle Optimization (MGEO) is a metaheuristic algorithm that uses fewer population vectors and control parameters, which makes it easy to use. The algorithm is inspired by the natural hunting strategy of golden eagles. The algorithm is known for its ability to adjust to high-dimensional space and perform effective global searches. The method provides better performance than other metaheuristic methods, especially cluster formation.

The MGEO algorithm works in two phases: the clustering and the refinement phases. In clustering phase, the vehicles in the network topology are grouped in to clusters based on factors such as signal strength and distance between vehicles which totally depends on four operations including hunting, reproduction, migration, and group formation. The proximity is determined by measuring the separation and signal strength between the vehicles. This phase involves four operators: hunting, group formation, reproduction, and migration. In the refinement phase, the clusters which are formed initially during clustering phase are further improved to improve cluster quality. This phase contains two search operations: local and global search. The local search improves cluster quality adjusting vehicles position. The global search improves the performance of overall clustering by repositioning clusters across the network.

To enhance the cluster quality, the clustering found in the first phase is improved in the refinement phase. Local and global search are the two operators used in this phase. The local search operator focuses on improving the cluster quality by shifting the members of the clusters within the cluster. The global search operator, on the other hand, focuses on improving the overall clustering quality by moving the clusters around in the network. MGEO algorithm is designed to improve the energy efficiency of the network by minimizing the distance between the vehicles in a cluster, thereby reducing communication energy consumption. The algorithm also aims to maximize cluster stability by minimizing the number of cluster head changes during the operation of the network. As previously stated, every golden eagle records the best place it has ever been.

Equation (1) describes the eagle hunting for good food and capturing prey for an attack. It can be used to calculate the optimal function of the golden eagle as follows.

1 $${\vec{S} _h}=\overrightarrow Z _{d}^{*} - {\vec{Z}} _{u}^{\prime }$$

Where${\vec{S}} _{h}$ denotes Eagle h’s attack vector, ${\vec{Z}}_{d} ^{*}$ indicates the best to date location visited by Eagle d, and ${\vec{Z}} _{u}^{\prime }$is the Eagle u’s current position.

Equation (2) shows the equation of hyperplane in dimensional space.

2 $${g_1}{z_1}+{g_2}{z_2}+ \cdots +{g_m}{z_m}=F \Rightarrow \sum\limits_{{h=1}}^{m} {{g_h}{z_h}=F}$$

Where$\vec {G}=\left[ {{g_1},{g_2},..,{g_m}} \right]$is the normal vector, $\vec {Z}=\left[ {{z_1},{z_2},..,{z_m}} \right]$is the variable vector, and $\vec {P}=\left[ {{p_1},{p_2},..,{p_m}} \right]$is the arbitrary point and $F={{*}}\mathop \sum \nolimits_{{h=1}}^{m} {g_h}{p_h}.$.

Equation (3) determines the demonstration of using a hyperplane in a golden eagle cruise vector and hyperplane normal${\vec {S}_h}$.

3 $$\sum\limits_{{k = 1}}^{j} {s_{k} z_{k} } = \sum\limits_{{k = 1}}^{g} {s_{k}^{r} z^{*} _{h} }$$

Where ${\vec {S}_h}$= [${s_1},{s_2},..,{s_m}$] is the attack vector, $\vec {Z}=\left[ {{z_1},{z_2},..,{z_m}} \right]$is the variable vector, and ${\vec {Z}^*}=[{z^*}_{1},{z^*}_{2},..,{z^*}_{m}]$is the selected prey location.

Equation (4) determines the value of the fixed variable.

4 $${V_j}=\frac{{F - \mathop \sum \nolimits_{{k,k \ne j}} {s_k}}}{{{s_j}}}$$

Where ${V}_{j}$is the Jth element of the destination V, *s*_*k*_ is the kth element of${\vec{S}}_{h}$, F indicates the right-hand side of (2), *s*_*j*_ is the Jth element of ${\vec{S}}_{h}$, and j is the fixed, variable index.

Equation (5) displays the cruise hyperplane’s general representation of the destination point as follows

5 $${\vec {V}_u}=\left( {{V_1}=random,\,{V_2}=random, \ldots {V_j}=\frac{{F - \mathop \sum \nolimits_{{k,k \ne j}} {s_k}}}{{{s_j}}} \ldots ,{X_m}=random} \right)$$

Equation (6) characterize the step vector for the brilliant falcon as

6 $$\Delta {z_u}=\overrightarrow {{e_1}} {o_s}\frac{{\overrightarrow {{S_u}} }}{{||\overrightarrow {{S_u}} ||}}+\overrightarrow {{e_2}} {o_x}\frac{{{{\vec {V}}_u}}}{{||\overrightarrow {{V_u}} ||}}$$

Where ${o_s}$ is the attack coefficient, ${o_x}$ is the cruise coefficient, $\overrightarrow {{e_1}}$ and $\overrightarrow {{e_2}}$ are random vectors lying in the interval [0, 1], $||{\mathrm{~}}\overrightarrow {{S_u}} ||$ and $||{\mathrm{~}}\overrightarrow {{V_u}} ||$ represents the Euclidean norms of attack and cruise vectors calculated in (7)

7 $$||\overrightarrow {{S_u}} ||=\sqrt {\mathop \sum \limits_{{k=1}}^{m} s_{k}^{2},} \quad ||\overrightarrow {{V_u}} ||=\sqrt {\mathop \sum \limits_{{k=1}}^{m} V_{k}^{2}}$$

Equation (8) determines the position of the golden eagles in an iteration *r* + 1 can be determined by simply adding their positions to the step vector in iteration r.

8 $${z^{r+1}}={z^r}+\Delta z_{u}^{r}$$

Equation (9) displays the linear transition used to calculate intermediate values.

9 $$\left\{ {\begin{array}{*{20}{c}} {{o_s}=o_{s}^{0}+\frac{r}{R}\left| {o_{s}^{R} - o_{s}^{0}} \right|} \\ {{o_v}=o_{v}^{0} - \frac{r}{R}\left| {o_{v}^{r} - o_{v}^{0}} \right|} \end{array}} \right.$$

The working of MGEO algorithm-based clustering can be summarized as Algorithm 1.

**Algorithm 1:**
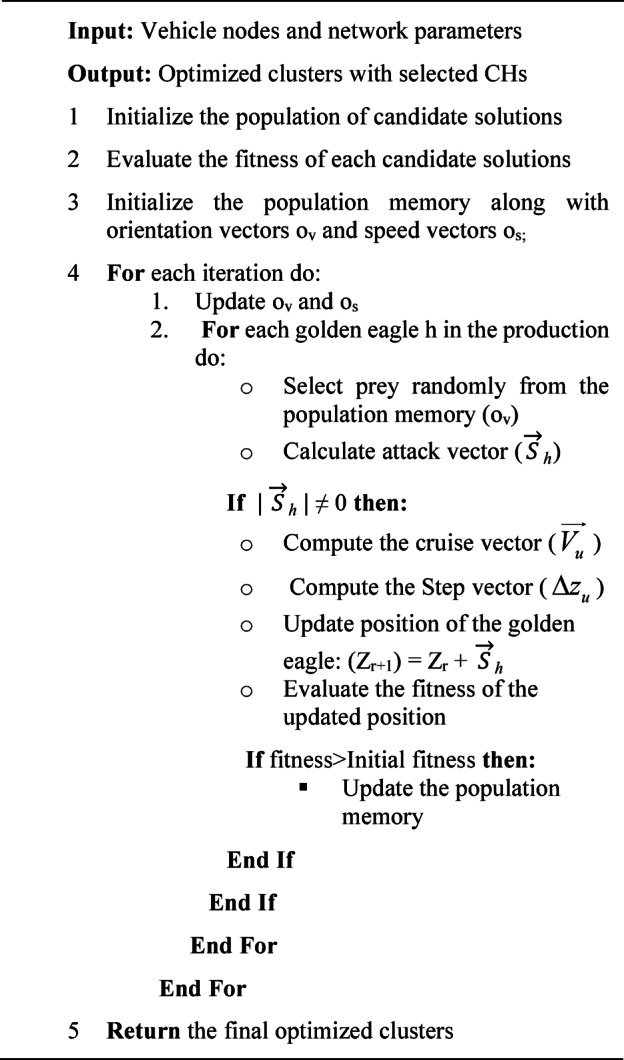
Formation of clusters using MGEO.

### Selection of CHs using improved Jackal optimization

After clustering, we gather important data from each vehicle node to decide on the CH. Finding the best option to reduce energy consumption is the goal of the CH selection process, which is approached as a single objective optimization problem. Taking into account variables like energy usage, network lifetime, congestion rate, data delivery rate, and drop rate, the CH gathers data from multiple locations throughout the network. An essential metric for evaluating the significance of information collection and transmission procedures is the energy model of the sensor location.

The IJO algorithm uses a set of mathematical operators, such as crossover, mutation, and selection, to generate new candidate solutions in each iteration. These operators are applied to the best-fit solutions to produce new offspring that can better meet design constraints.

The initialization of Jackal is expressed in (10)

10 $$~~{T_0}=Low+~rand*\left( {Up - Low} \right)$$

Where Low and Up represents the lower and upper limit of the region, rand represents the uniform number lies inside [0, 1].

The victim location matrices are generated in the initial step at random as follows in (11).

11 $${\rm{Prey}} = \left[ {\begin{array}{*{20}{c}} {{T_{1,1}}}& \ldots &{{T_{1,h}}}& \cdots &{{T_{1,m}}} \\ {{T_{2,1}}}& \cdots &{{T_{2,h}}}& \ldots &{{T_{2,m}}} \\ \cdots & \cdots & \cdots & \cdots & \cdots \\ \vdots & \vdots & \vdots & \vdots & \vdots \\ {{T_{M - 1,1}}}& \cdots &{{T_{M - 1,h}}}& \cdots &{{T_{M - 1,m}}} \\ {{T_{M,1}}}& \cdots &{{T_{M,h}}}& \cdots &{{T_{M,m}}} \end{array}} \right]$$

Where M indicates the number of victims and n indicates the number of measurements.

Equations (12) and (13) determines the Golden Fox Hunt’s mathematical model and is defined as follows (|W| > 1):

12 $${T_1}(r)={T_N}(r) - W \cdot \left| {{T_N}(r) - ek.prey(r)} \right|$$

13 $${T_2}(r)={T_{DN}}(r) - W \cdot \left| {{T_{DN}}(r) - ek.prey(r)} \right|$$

Where r is the current iteration ${T_N}(r)$ and ${T_{DN}}(r)$ indicates the male and female jackal positions, prey(r) indicates the prey’s position vector, ${T_1}(r)$ and ${T_2}(r)$ represents the updated positions of male and female jackals, the distance between jackal and prey are represented as $\left| {{T_N}(r) - ek.prey(r)} \right|$ and $\left| {{T_{DN}}(r) - ek.prey(r)} \right|$.

Equations (14), (15), and (16) indicates the prey’s energy, and it is calculated as follows:

14 $$W={W_1} \cdot {W_0}$$

15 $${W_1}={X_1} \cdot \left( {1 - \left( {r/R} \right)} \right)$$

16 $${W_0}=2{\mathrm{*r'-1}}$$

Where W represents the prey’s Evading energy, *W*_1_ is the prey’s decreasing energy, *W*_0_ is the prey’s initial state energy, r′ indicates an arbitrary number between [0, 1],

*X*_1_ is a constant value equal to 1.5, r is the current iteration, and R indicates the maximum number of iterations. R is a random number vector calculated using the Levy flight function, and (17) represents the distance between the golden fox and the prey.

17 $$ek=0.05=KD(T)$$

Where ek indicates the vector of random based on Levy distribution, and KD is the levy flight function which is calculated.

18 $$KD(T)=0.01 \times \frac{{(\eta \times \rho )}}{{(|{\kappa ^{(1/\delta )}}|)}};\rho ={\left\{ {\frac{{\Omega (1+\delta ) \times {\mathrm{Sin}}(\Delta \delta /2)}}{{\Omega \left( {\frac{{1+\delta }}{2}} \right) \times \delta \times \left( {{2^{\frac{{\delta - 1}}{2}}}} \right)}}} \right\}^{1/\delta }}$$

Where ⸹ is a default constant equals to 1.5.

Equation (19) displays the updated hunting position for male and female golden foxes is T(*r* + 1). The dodge power decreases as the golden foxes pursue their prey.

19 $$T(r+1)=\frac{{{T_1}(r)+{T_2}(r)}}{2}$$

20 $${T_1}(r)={T_N}(r) - W \cdot |ek.{T_N}(r) - prey(r)|$$

21 $${T_2}(r)={T_{DN}}(r) - W \cdot |ek.{T_{DN}}(r) - prey(r)|$$

The working of IJO algorithm-based clustering can be summarized as Algorithm 2.

**Algorithm 2:**
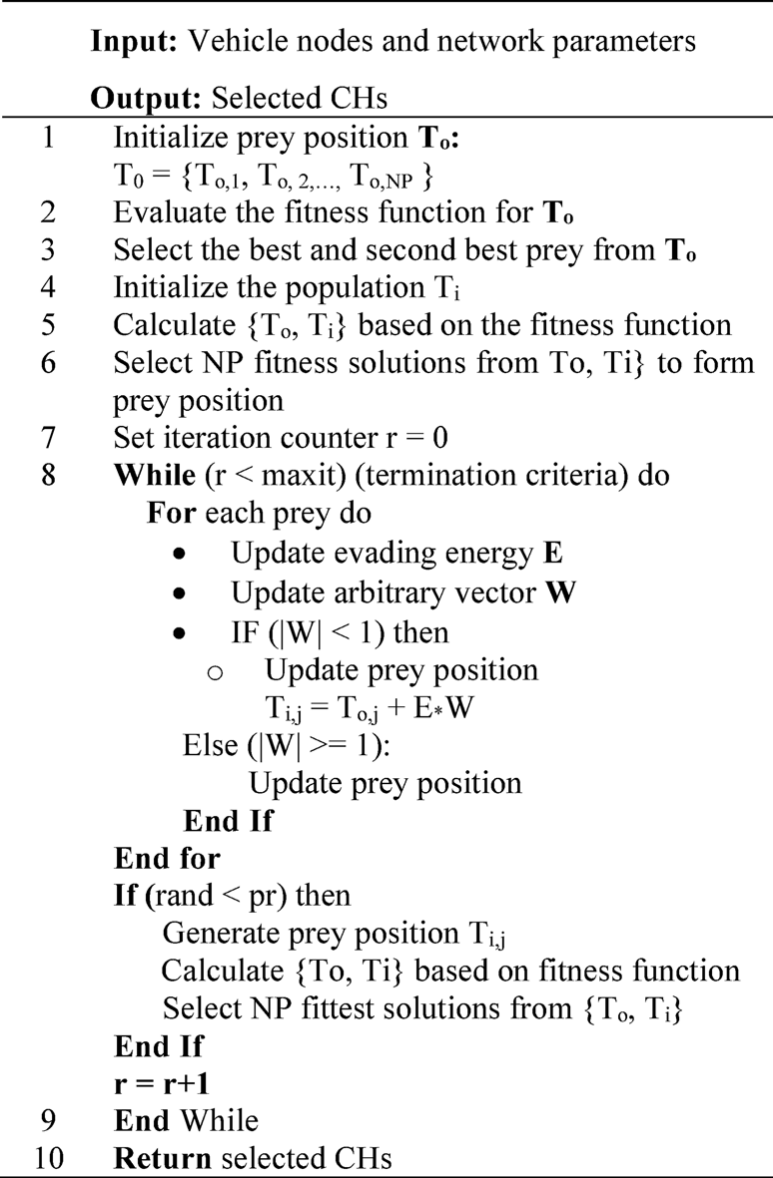
Selection of CH using IJO.

### Data dissemination between vehicle to infrastructure (V2I)

Following CH selection and identification of neighboring nodes for Vehicle to Vehicle (V2V) data routing, data dissemination to RSUs is crucial in V2I communication. Using a MONN optimal RSUs are selected for efficient data routing. MONN learns from input features such as distance, signal strength, vehicle location, and traffic volume.

**Fig. 3:**
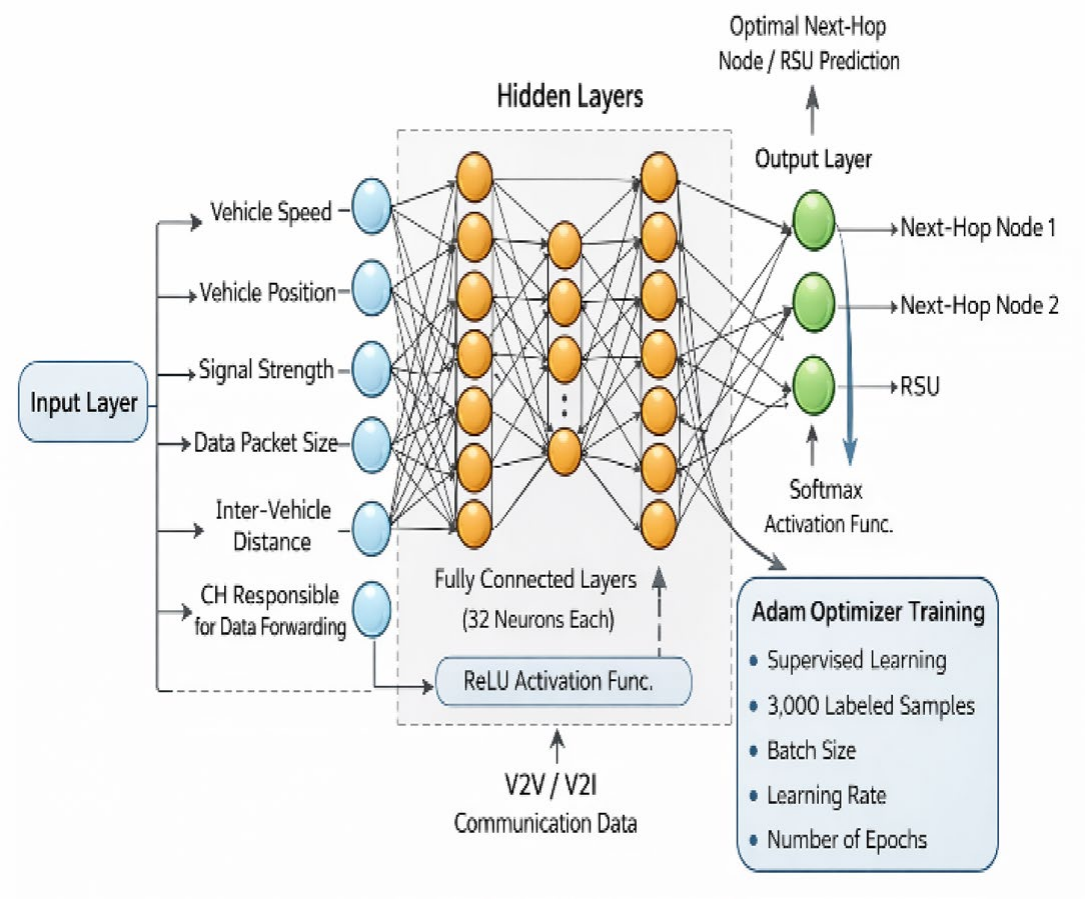
Architecture of MONN for Data dissemination between V2I.

Training MONN model is performed using input-output pairs derived from the NS2 simulation environment, enabling it to predict the best RSUs and next hop paths for efficient data dissemination. The architecture of MONN for V2I communication is shown in Fig. 3.

1. In the V2I communication scenario, the input layer receives data, including information about the vehicle speed, position, signal strength, data packet size, inter vehicle distance and the CH responsible for data forwarding.
2. The hidden layer consists of two fully connected layers with 32 neurons each, which process the input data through nonlinear transformations using the ReLU activation function. This enables the network to learn high-dimensional feature representation. This feature then fed into the output layer.
3. The **output layer**, equipped with a Softmax activation function, predicts the optimal next-hop node or RSU for data routing. Supervised learning is employed to train the MONN model using a labeled dataset of 3,000 samples, where each sample maps the input features to the corresponding optimal routing decision.
4. The Adam optimizer trains the model with experimentally selected parameters including batch size, learning rate and number of epochs. The trained model processes real-time V2V and V2I communication data to determine the next-hop node during the dissemination process.

Equation (22) describes the operation of the input layer

22 $${p_K}=\mathop \sum \limits_{{j=1}}^{J} \left( {i{Z_{r,j}}{N_{in}}+{y_{1\left( r \right)}}} \right)$$

Where J is the quantity of input data (j = 1, 2, 3⋯K), $i{Z_{r,j}}$is the weights coefficient between input and hidden layers, ${N_{in}}$is an input variable, and ${y_{1(r)}}$ is the corresponding bias to the neuron in the hidden layer. The hidden layer neuron is subjected to the transfer function K after summing the compensation value and the weighted inputs of multiple connections.

Equation (23) describes the outputs of the hidden layer and is calculated as follows:

23 $${x_K}=f\left( {\mathop \sum \limits_{{j=1}}^{J} \left( {i{Z_{r,j}}{N_{in}}+{y_{1\left( r \right)}}} \right)} \right)$$

Where f is the initiation capability. ${x_K}$ is the output of the hidden layer and feeds neurons. The output layer of the neuron ${B_j}$undergoes the same process.

Equation (24) describes the output of the neuron in the output layer and is calculated as follows:

24 $${B_j}=f\left( {\mathop \sum \limits_{{s=1}}^{R} \left( {l{Z_{r,L}}{x_K}+{y_{2\left( L \right)}}} \right)} \right)$$

Where ${B_j}$is the neuron’s output signal, $l{Z_{r,L}}$is the weights coefficient between hidden and output layers, and ${y_{2\left( L \right)}}$ is the corresponding bias to the neuron in the output layer.

It is essential to note that normalizing each input variable before training the neural network is convenient. For improved efficiency, training begins with a period of nonlinear transfer functions. The information standardization strategy is regularly refined utilizing straight planning called the min-max scale. The information factors are standardized utilizing the accompanying condition.

25 $${n_{I,P}}=\frac{{\left( {{N_{in}} - {N_{{\mathrm{min}}}}} \right)}}{{{N_{{\mathrm{max}}}} - {N_{{\mathrm{min}}}}}}\left( {{B_{{\mathrm{max}}}} - {B_{{\mathrm{min}}}}} \right)+{B_{{\mathrm{min}}}}$$

Where ${n_{I,P}}$ is the normalized variable, N_in_ is the variable before normalization, N_min_ and N_max_, B_min_ and B_max_ are the minimum and maximum variables before after normalization, respectively.

When MONN was used for data distribution between conventional V2I methods, the data transfer ratio, final delay, and throughput all significantly improved, demonstrating MONN’s potential as a potent tool for improving the efficacy and efficiency of V2I communication in VANETs. Algorithm 3 provides a summary of the MONN’s data dissemination working steps.

**Algorithm 3:**
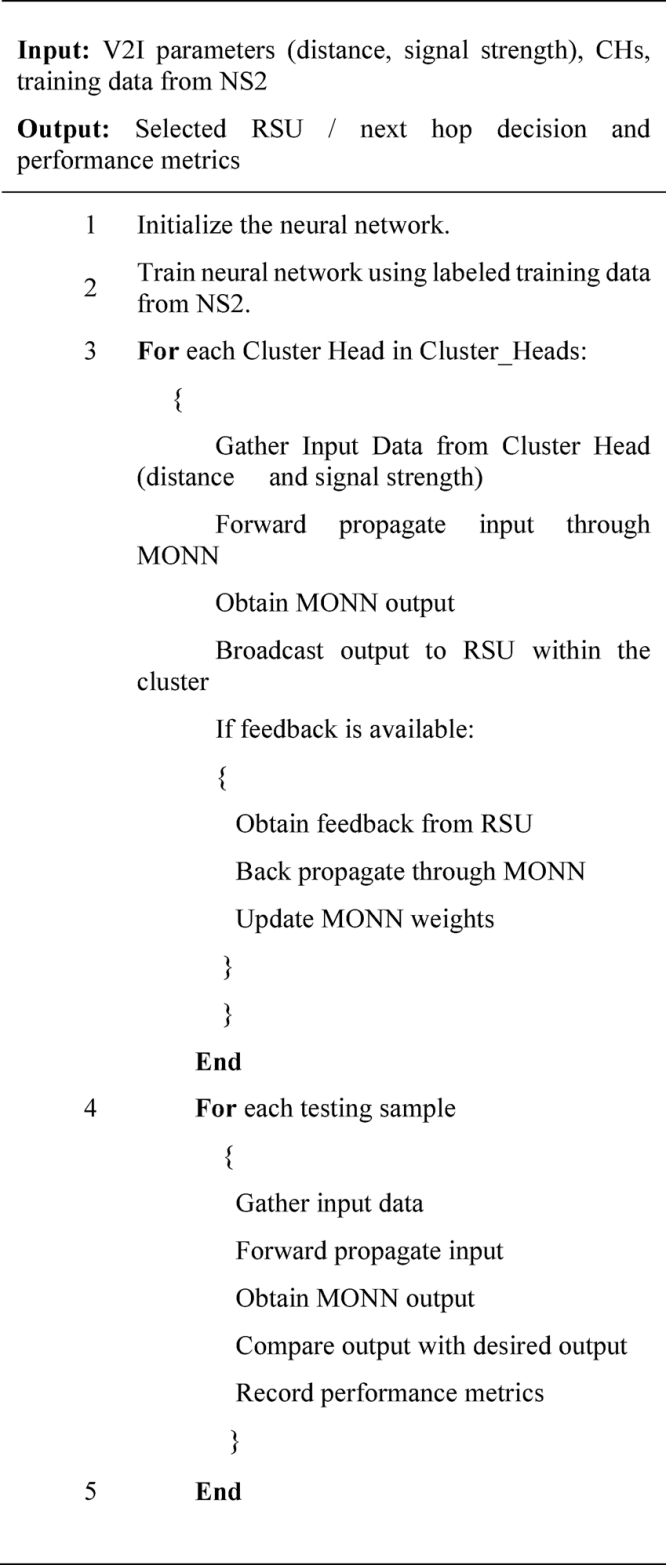
Data dissemination between V2I.

## Results and discussion

This section evaluates and compares the proposed HMM OCR approach with the existing VANET routing methods. We conduct tests in two distinct scenarios: (i) varying simulation duration to evaluate routing stability under dynamic VANET conditions, and (ii) varying vehicle density to evaluate scalability. The NS2 simulator is used to assess VANET performance, for all routing tests.

The simulation results are compared to traditional routing protocols such as P-AOMDV, DSR, Q-AODV, and RRIN are compared with the simulation results of the suggested HMM-OCR method. To demonstrate the effectiveness of the proposed method, the results were examined using a variety of performance metrics.

**Fig. 4:**
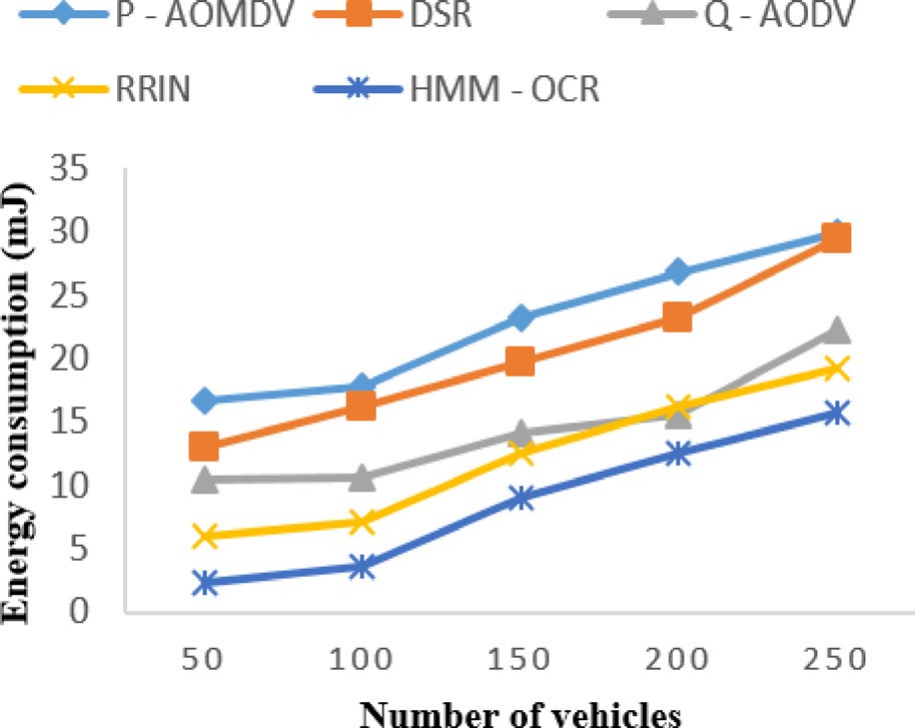
Energy consumption with varying vehicles.

For instance, in the case of energy consumption, the percentage difference for HMM-OCR compared to RRIN is 86.07%, 66.55%, 33.15%, 24.82%, and 20.38% for 50, 100, 150, 200, and 250 vehicles. The results shown in Fig. 4 indicate that our HMM-OCR method is more energy-efficient than existing state-of-the-art methods for VANETs with varying numbers of vehicles.

**Fig. 5:**
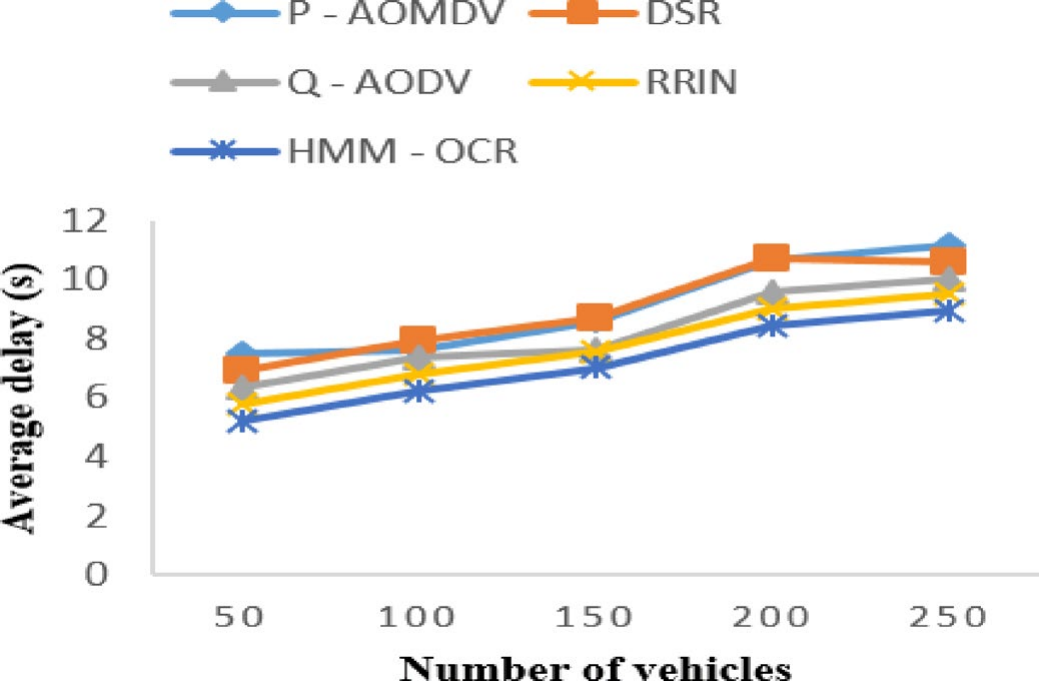
Average delay with varying vehicles.

For instance, with 50 vehicles, the average delay for HMM-OCR is 5.236 s, which is lower than all other methods by a significant margin. From Fig. 5, we can see that the HMM-OCR method decreases the average delay compared to all other methods.

### Setting up the simulation

The performance of the proposed model was evaluated through extensive simulations. Table 1 shows the summary of simulation parameters.

**Table 1:** Summary of simulation setup.

Parameter	Value
Network size	1000 m*1000m
Number of vehicles	50, 100, 150, 200, 250
Size of beacon	20 byte
MAC	IEEE 802.11p
Propagation model	Two-ray ground
Traffic model	Constant bit rate
Transmission range	250 m
Bandwidth	2 Mbps
Size of packet	512 bytes
Interval of beacon	200 ms
Capacity of channe	18 Mbps
Frequency	5.89 GHz
Transmission power	15mW
Network	VANET
Simulator	NS2

### Selection of baseline protocol and justification

The widely used VANET routing protocols P-AOMDV, DSR, Q-AODV, and RRIN were chosen as baseline techniques to assess the efficacy of the suggested HMM-OCR scheme. These protocols allow for a thorough comparative analysis by representing various routing philosophies and optimization techniques frequently employed in vehicular ad hoc networks.

- Multiple loop-free and link-disjoint paths between source and destination are maintained by P-AOMDV, a multipath extension of AODV. It is selected to symbolize conventional multipath routing techniques that do not use clustering or intelligent optimization mechanisms and instead concentrate on path redundancy to increase reliability.
- DSR is a traditional reactive routing protocol that uses route caching and source routing. In highly dynamic VANET environments, it serves as a baseline to assess the shortcomings of traditional on-demand routing schemes, especially with regard to scalability and routing overhead.
- By using Q-learning to enhance routing choices based on network feedback, Q-AODV incorporates reinforcement learning into the AODV framework. This protocol is chosen to illustrate learning-based routing techniques so that the suggested hybrid intelligence-driven HMM-OCR framework and traditional learning mechanisms can be compared.
- RRIN is a routing protocol for VANETs that takes link stability and node reliability into account, making it appropriate as a representative reliability-aware routing scheme. It does not make use of clustering or metaheuristic optimization, but it does partially address dynamic topology problems.

The simulation results for the HMM-OCR method and the current cutting-edge routing techniques, P-AOMDV, DSR, Q-AODV, and RRIN, for various vehicle densities are displayed in Table 2. The performance outcomes in the table carried with the same node densities and identical network configurations. To ensure a fair and unbiased comparison, all of the routing algorithms used the same simulation parameters including node traffic configuration, density, mobility model, and simulation time. The “Difference” column denotes the improvement achieved by HMM-OCR over the best-performing baseline protocol under the same conditions.

**Table 2:** Results in comparison with respect to varying densities.

	Nodes	Routing algorithms					
		P-AOMDV^15^	DSR^16^	Q-AODV^17^	RRIN [14]	HMM-OCR	Difference
Energy consumption (mJ)	50	16.596	13.036	10.476	5.916	**2.356**	**86.07%**
	100	17.809	16.249	10.689	7.129	**3.569**	**66.55%**
	150	23.196	19.636	14.076	12.516	**8.956**	**33.15%**
	200	26.803	23.243	15.516	16.123	**12.563**	**24.82%**
	250	29.927	29.367	22.202	19.247	**15.687**	**20.38%**
Average delay (S)	50	7.476	6.916	6.356	5.796	**5.236**	**0.56**
	100	8.475	7.915	7.355	6.795	**6.235**	**0.56**
	150	9.263	8.703	7.602	7.583	**7.023**	**0.56**
	200	10.696	10.701	9.576	9.016	**8.456**	**0.56**
	250	11.172	10.612	10.052	9.492	**8.932**	**0.56**
Delivery ratio (%)	50	76.618	80.854	85.09	91.326	**97.562**	**6.236**
	100	74.389	77.435	84.651	89.762	**95.123**	**5.361**
	150	69.491	74.527	75.563	82.999	**90.235**	**7.236**
	200	68.181	72.417	80.653	84.945	**89.125**	**4.18**
	250	63.042	68.328	78.564	81.256	**85.036**	**3.78**
Drop ratio (%)	50	23.3	18.064	12.828	7.592	**2.356**	**5.236**
	100	26.84	24.764	14.052	12.423	**5.896**	**6.527**
	150	30	21.604	19.528	15.192	**9.056**	**6.136**
	200	32.513	27.277	22.041	17.901	**11.569**	**6.332**
	250	39.508	32.272	28.026	21.8	**17.264**	**4.536**
Routing overhead (%)	50	33.81	28.11	19.22	18.72	**11.02**	**7.7**
	100	37.508	32.66	26.96	21.19	**15.57**	**5.62**
	150	41.14	40.657	26.23	25.04	**18.35**	**6.69**
	200	44.357	38.77	34.69	26.266	**21.569**	**4.69**
	250	46.356	40.659	34.962	29.356	**23.568**	**5.78**
Throughput (kbps)	50	3.12	3.30	3.47	3.73	**3.98**	**21.95%**
	100	3.03	3.16	3.45	3.66	**3.88**	**18.21%**
	150	2.84	3.04	3.08	3.39	**3.68**	**17.43%**
	200	2.78	2.95	3.29	3.47	**3.64**	**14.70%**
	250	2.57	2.79	3.21	3.31	**3.47**	**12.91%**

Bold values indicate the best results achieved by the proposed HMM-OCR method. Additionally, the table highlightsthe difference between the proposed results and the best existing values for each metric.

**Fig. 6:**
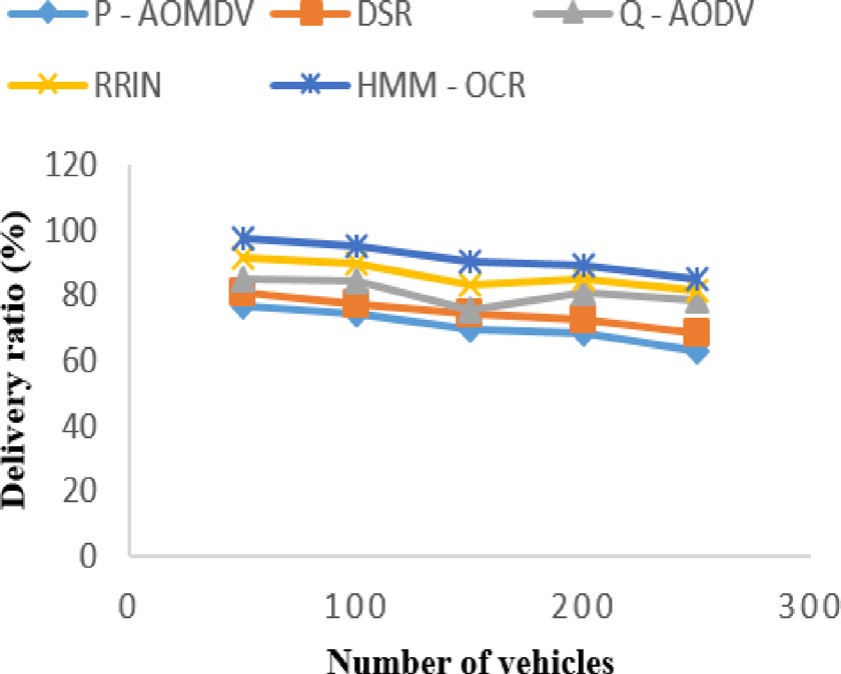
PDR with varying vehicles.

The delivery ratio of the proposed HMM-OCR method outperformed all other existing methods at all vehicle densities. As the number of vehicles increased from 50 to 250, P-AOMDV, DSR, Q-AODV, RRIN, and HMM-OCR methods showed a decrease in delivery ratio. However, the HMM-OCR method maintained the highest delivery ratio of 97.562%, 95.123%, 90.235%, 89.125%, and 85.036% for 50, 100, 150, 200, and 250 vehicles, respectively. Figure 6 indicates that the proposed method can ensure reliable data delivery even in dense traffic scenarios.

**Fig. 7:**
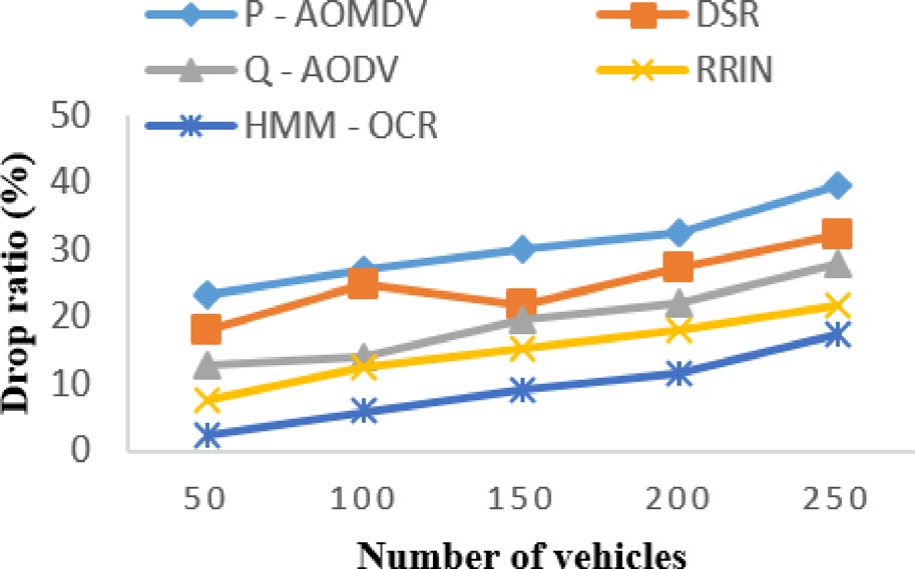
Drop ratio with varying vehicles.

The analysis of Fig. 7 reveal that the drop ratio increases with an increase in the number of vehicles for all routing methods, and it is expected since the higher the number of vehicles, the more congested the network becomes, leading to packet drops. The proposed HMM-OCR method outperforms all the existing routing methods regarding drop ratio. For example, the drop ratio for HMM-OCR is only 2.356%, at 50 vehicles, but for P-AOMDV, it is 23.3%.

**Fig. 8:**
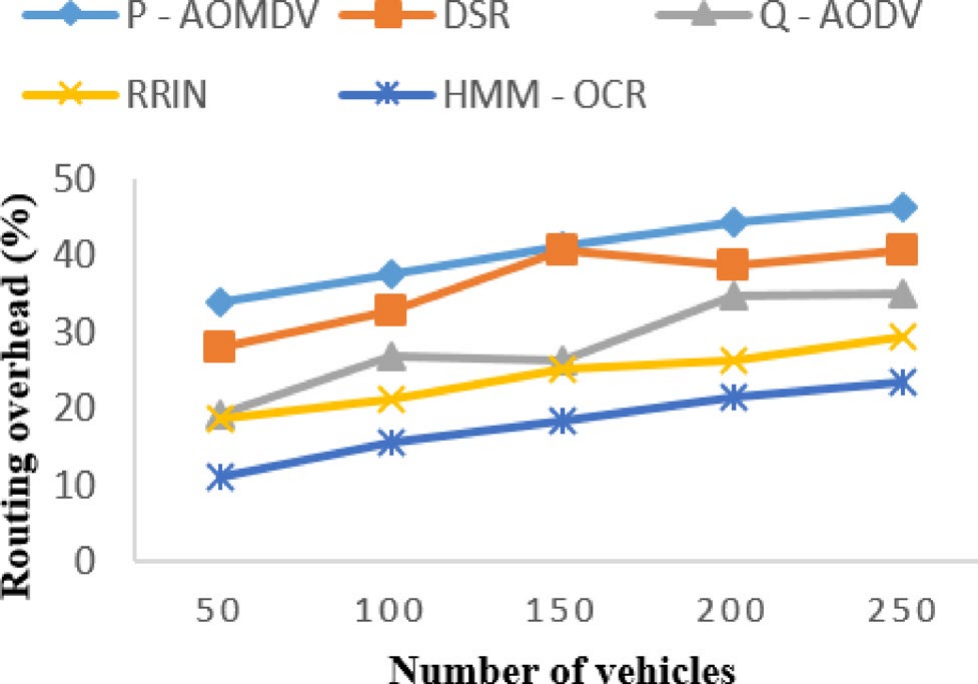
Routing overhead with varying vehicles.

It can be observed that HMM-OCR has the lowest routing overhead compared to other routing methods. The routing overhead of HMM-OCR for 50, 100, 150, 200, and 250 vehicles are 11.02%, 15.57%, 18.35%, 21.569%, and 23.568%, respectively. It can be seen that as the number of vehicles increases, the routing overhead of all the routing methods increases as well. From Fig. 8, we can see that HMM-OCR performs the best in routing overhead, making it an efficient routing method for VANET.

**Fig. 9:**
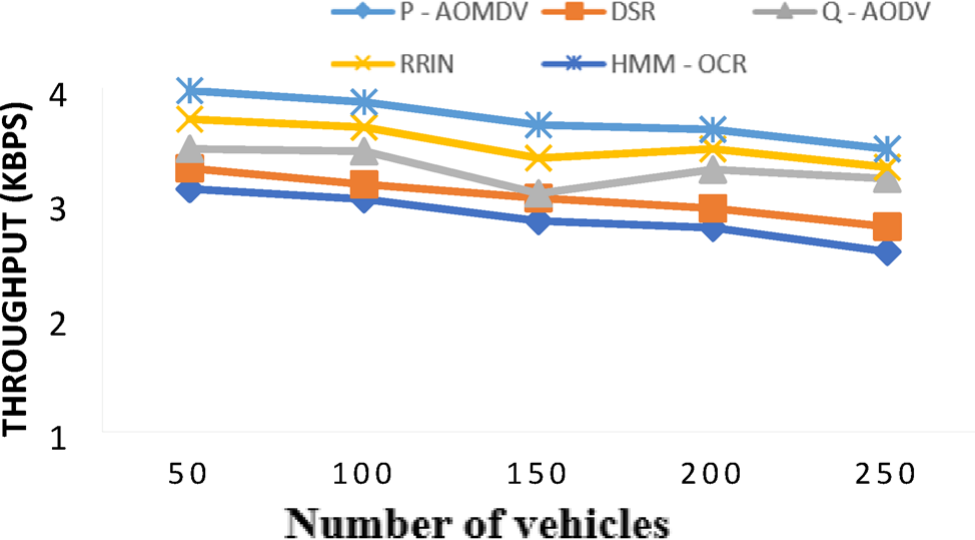
Throughput with varying vehicles.

In the case of throughput, the percentage improvement achieved by HMM-OCR over P-AOMDV, DSR, Q-AODC, AND RRIN for 50 vehicles is 27.56%, 20.61%, 14.69%, and 6.70% respectively. Similarly, for 100 vehicles, HMM-OCR improves throughput respectively. The results shown in Fig. 9 shows that HMM-OCR has a higher throughput than other routing protocols no matter about vehicle density on the road. This shows that it is a good wat to improve efficient data delivery in urban scenarios.

## Conclusion

The HMM-OCR method for optimized cluster-based routing in VANETs shows promising results in improving communication and coordination among vehicles, thereby enhancing the safety and efficiency of transportation. The MGEO algorithm used for clustering and the IJO algorithm for CH selection ensure energy-efficient data dimensions and take into account several design constraints. E2E routing between the source and destination is ensured by the MONN used for data dissemination with infrastructure. This study, therefore, contributes to the development of more reliable and effective routing protocols for VANETs, potentially enhancing road safety and transportation quality. The proposed strategy can basically make cars interact better, traffic flow more smoothly, and accidents happen less often. All of these things will be good for the ecosystem around cars and trucks.

For example, the proposed HMM-OCR uses 20.38%, 34.38%, 60.72%, and 62.43% more energy than RRIN, Q-AODV, DSR, and P-AOMDV for 250 vehicles. The average delay for 250 cars is 0.56 s, 1.12 s, 1.68 s, and 2.24 s. For 250 vehicles, the PDR difference is 3.78%, 6.47%, 16.7%, and 21.9%. For 250 vehicles, the packet drop ratio difference is 4.53%, 10.76%, 15%, and 22.24%, and the routing overhead difference is 5.87%, 11.395, 17.09%, and 22.87%. The throughput is 34.98%, 24.37%, 8.10%, and 4.83% lower for 250 vehicles than it is for P-AOMDV, DSR, Q-AODV, and RRIN, respectively.

The HMM-OCR method is important in making VANET communication safer and more efficient. The proposed framework improves coordination among vehicles, reduce energy consumption, and enhances routing reliability, but it could be even better to deal with problems that come up in the real-world. Future work should focus on security issues, scalability for large networks, and adaptability to different traffic conditions. In should also include multi-objective optimisation, real-world validation, and new AI-driven technologies to improve communication.

## Data Availability

The datasets used and/or analyzed during the current study are available from the corresponding author on reasonable request.
